# MicroRNA Let-7d-3p Contributes to Cardiac Protection via Targeting HMGA2

**DOI:** 10.3390/ijms20071522

**Published:** 2019-03-27

**Authors:** Lee Lee Wong, Eng Leng Saw, Jia Yuen Lim, Yue Zhou, Arthur Mark Richards, Peipei Wang

**Affiliations:** 1 Cardiovascular Research Institute, National University Heart Centre, Singapore 117599, Singapore; mdcwll@nus.edu.sg (L.L.W.); sawengleng@gmail.com (E.L.S.); kayla.ljy@gmail.com (J.Y.L.); mdczhouy@nus.edu.sg (Y.Z.); mark.richards@nus.edu.sg (A.M.R.); 2 Department of Medicine, Centre for Translational Medicine, Yong Loo Lin School of Medicine, National University of Singapore, Singapore 117599, Singapore; 3 Christchurch Heart Institute, Department of Medicine, University of Otago, Christchurch 8014, New Zealand

**Keywords:** microRNA, cardiac protection, apoptosis, Let-7d-3p, HMGA2

## Abstract

We tested the hypothesis that Let-7d-3p contributes to cardiac cell protection during hypoxic challenge. Myoblast H9c2 cells and primary neonatal rat ventricular cardiomyocytes (NRVM) were transfected with five selected miRNA mimics. Both cell lines were subjected to 0.2% oxygen hypoxia. The protective effects of these miRNAs were determined by assessment of cell metabolic activity by CCK8 assay and measurement of lactate dehydrogenase (LDH) release as a marker of cell injury. Apoptosis and autophagy flux were assessed by Annexin V/7-AAD double staining and the ratio of LC3 II/I with Baf-A1 treatment, an autophagy flux inhibitor, respectively. Luciferase-reporter assay, RT-qPCR and Western blots were performed to identify the changes of relevant gene targets. Among five miRNA mimic transfections, Let-7d-3p increased CCK8 activity, and decreased LDH release in both H9c2 and NRVM during hypoxia. Apoptosis was significantly reduced in H9c2 cells transfected with Let-7d-3p mimic. Autophagy and autophagy flux were not affected. In silico, mRNAs of HMGA2, YY1, KLF9, KLF12, and MEX3C are predicted targets for Let-7d-3p. Luciferase-reporter assay confirmed that Let-7d-3p bound directly to the 3’-UTR region of HMGA2, MEX3C, and YY1, the down-regulations of these mRNAs were verified in both H9c2 and NRVM. The protein expression of HMGA2, but not others, was downregulated in H9c2 and NRVM. It is known that HMGA2 is a strong apoptosis trigger through the blocking of DNA repair. Thus, we speculate that the anti-apoptotic effects of Let-7d-3p mimic during hypoxia challenge are due to direct targeting of HMGA2.

## 1. Introduction

Circulating microRNAs (miRNAs) were first detected in human plasma a decade ago. They have since been widely measured in both serum and plasma as promising biomarkers for various diseases. Discovery of therapeutic miRNA targets through dysregulated plasma miRNAs observed in clinical cohorts has been a productive strategy. Well-studied miRNAs in cardiovascular disease include miR-208a, miR-208b, and miR-499 which are known as potential biomarkers for acute myocardial infarction [1,2]. An antimir of miR-208a significantly suppressed myocardial hypertrophy and perivascular fibrosis in rats fed a high-salt diet [3] and miR-499 decreased apoptosis during ischemia/reperfusion [4]. The miR-208 family has displayed its potential to provide both biomarkers and therapeutic targets. We aimed to explore the therapeutic potential of miRNAs identified from clinical studies.

We recently reported the potential of using miRNAs as biomarkers from a total of 1710 non-heart failure controls and heart failure patients [5]. We screened ~200 circulating miRNAs and identified a set of dysregulated miRNAs in heart failure patients. Based on higher fold change and higher area under the curve (AUC), and also the homologues of miRNA sequences between human and rat, five of these dysregulated miRNAs were selected for functional study in an ex vivo hypoxia model. Let-7d-3p, miR-503-5p and miR-134-5p were significantly upregulated, and miR-374b-5p and miR-30b-5p were significantly down regulated (*p* < 0.0001, after False Discovery Rate (FDR) correction) [5]. The upregulation of miR-503 promotes cardiac fibrosis in the transverse aortic constriction (TAC) mouse and Angiotension II treated cardiac fibroblast [6]. MiR-134 level is significantly higher in patients with acute myocardial infarction and this increase is strongly associated with mortality or development of heart failure [7]. Suppression of miR-30b transcription by E2F1 inhibited cardiomyocyte necrosis and reduced myocardial infarction size upon ischemia/reperfusion injury [8]. Overexpression of Let-7d (5p) attenuated collagen deposit, impeded cardiac fibrosis, and improved cardiac function in myocardial infarction mice via regulating the platelet-activating factor receptor (Ptafr) [9]. Thus, these results suggested it was worthwhile to investigate the possible therapeutic roles of these selected miRNAs in ischemic injury.

Lethal-7 or Let-7 was one of the first two known miRNAs discovered in Caenorhabditis elegans [10] and the first known human miRNA [11]. Let-7 family is comprised of 13 members that are highly conserved across species [12]. They share the same seed sequence and target multiple gene targets in various cell types. Many gene targets of the Let-7 family are known to play roles in ischemic heart disease, including TLR4, lox-1, Bcl-xl, and AGO1 [13]. Inhibition of Let-7c improved cardiac function after myocardial infarction through reducing cardiac fibrosis and apoptosis [14]. Restoring Let-7g protects cardiomyocytes from apoptosis via targeting Pik3ip1, an inhibitor of the Akt survival signaling pathway during acute ischemia reperfusion injury [15]. Transfecting Let-7d (5p) mimic into human carotid plaque ex vivo exerts an anti-inflammatory effect in a diabetes-associated atherosclerosis disorder [16]. Let-7d-3p (previous ID: Let-3d*) is the passenger strand and used to be considered as “non-functional”. Therefore, little is known about Let-7d-3p and there is no study in the setting of ischemia or in the cardiac cell line model.

Acute myocardial infarction, a primary cause of heart failure, is a leading cause of mortality worldwide. Cardiomyocytes have no regenerative capacity, therefore cardiac protection against ischemic injury is critical to salvage ischemic myocardium. Apoptosis and autophagy are two distinct processes activated concurrently in ischemia and/or ischemia reperfusion. They play key roles in mediating cardiomyocyte death. Apoptosis or programmed cell death is triggered during and after myocardial infarction and contributes to cardiomycyte cell death [17]. The level of apoptosis could affect infarct size and the extent of adverse left ventricular remodeling, potentially leading to heart failure after acute myocardial infarction [18]. Autophagy is activated by deprivation of nutrients and energy due to ischemia. It may be protective by removal of damaged mitochondria [19], restoring ATP production [20], and/or removal of toxic protein aggregates [21]. However, excessive autophagy induced by ischemia is detrimental [22,23]. The inhibition of ischemic-induced apoptosis and autophagy in the heart has been approved to be protective [24,25,26]. Therefore, in this study we investigated the effects of Let-7d-3p on apoptosis and autophagy and the underlying mechanisms. Genes of Mex3C, KLF9, KLF12, HMGA2, and YY1 are the in silico Let-7d-3p predicted targets related to cell survival, apoptosis, and cardiac functions. In particularly, HMGA2, a member of high mobility group A (HMG), plays a vital role in cardiogenesis [27]. Overexpression of HMGA2 induced apoptosis by upregulation of cleaved caspase 3 via DNA damage pathway [28]. However, the role of HMGA2 in cardiac protection via apoptosis and autophagy is yet to be determined.

Taking the approach of functional screening of dysregulated circulating miRNAs identified from heart failure patients, we discovered potential therapeutic miRNA targets for cardioprotection. Using an ex vivo hypoxia modelof H9c2 and NRVM against hypoxia, we demonstrated Let-7d-3p is a promising therapeutic miRNA target in cardiac ischemia.

## 2. Results

### 2.1. Protective Effect of Let-7d-3p in H9c2 and NRVM during Hypoxia Challenge

Five miRNAs of interest (Table 1) were selected for mimic transfection. The mature form of these miRNA sequences are conserved in both human and rat species (Table 1). Seed region sequences are highlighted.

**Figure 1:**
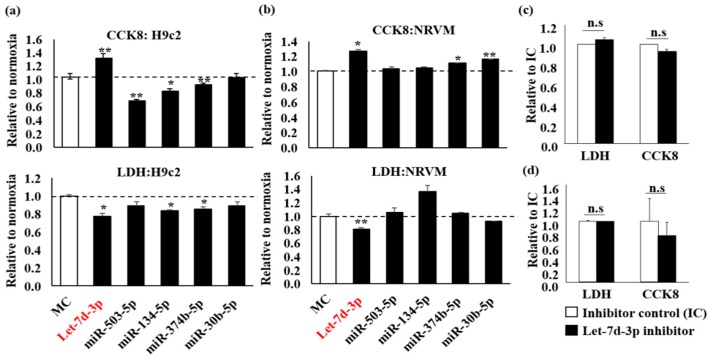
Functional screening of miRNA targets in H9c2, rat cardiomyoblast cells, and NRVM, neonatal rat ventricular cardiomyocytes, against hypoxia injury. Upon five miRNA mimic transfections, cell metabolic activity CCK8 and lactate dehydrogenase (LDH) release were measured in (**a**) H9c2, and (**b**) NRVM. With Let-7d-3p inhibitor transfection, CCK8 and LDH were measured in (**c**) H9c2 and (**d**) NRVM under hypoxia challenge. Experiments were repeated at least 3 times in triplicates. Data are presented as Mean ± SD with Student’s t-test, * *p* < 0.05, ** *p* < 0.01, n.s = not significant.

H9c2 cells were transfected with miRNA mimics and subjected to hypoxia, Let-7d-3p enhanced CCK8 activity and concomitantly decreased lactate dehydrogenase (LDH) release (Figure 1a, *p* < 0.05 vs. mimic control, MC). Parallel experiments were conducted in neonatal rat ventricular myocytes (NRVM). Let-7d-3p increased CCK8 and decreased LDH release as well (Figure 1b, *p* < 0.05 vs. MC). Since only Let-7d-3p showed a protective effect, we investigated the effect of Let-7d-3p inhibitor. There were no significant effects observed in both H9c2 (Figure 1c) and NRVM (Figure 1d). Thus, we postulated an important role for Let-7d-3p mimic in cardiac cells under hypoxic challenge.

### 2.2. The Protective Effect of Let-7d-3p Is Mediated via Apoptosis but Not Autophagy

**Figure 2:**
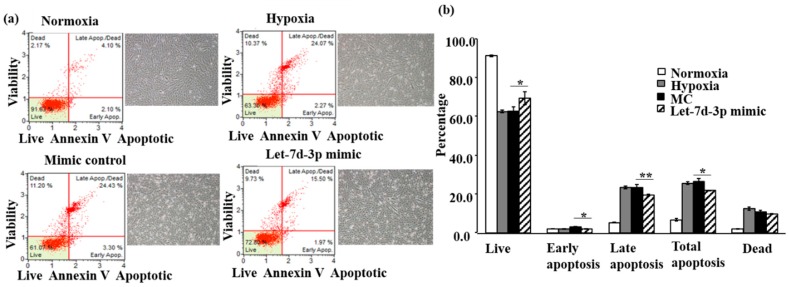
The protective effect Let-7d-3p is via the inhibition of apoptosis. With Annexin V/7-AAD double staining, Let-7d-3p increased live cells and decreased early and late apoptosis and total apoptosis cells in H9c2 subjected to hypoxia challenge. Results are shown in (**a**) Annexin-V MUSE cell analysis and morphology; (**b**) The percentage of cells in live, early apoptosis, late apoptosis, total apoptosis, and dead respectively. Experiments were repeated twice in triplicate, Data are presented as Mean ± SD with Student’s t-test, * *p* < 0.05, ** *p* < 0.01.

**Figure 3:**
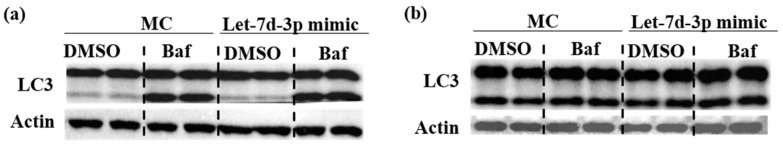
The protective effect of Let-7d-3p is not via the regulation of autophagy. Autophagy flux was accessed by the ratio of LC3 II/I with Bafilomycin A1 (Baf) treatment or Dimethyl sulfoxide (DMSO) for 2 h. Let-7d-3p did not induce any changes of LC3 II/I with or without Baf in (**a**) H9c2 (**b**) and NRVM.

To ascertain the protective mechanism, we assessed apoptosis using Annexin V/7-AAD double staining in H9c2. Total apoptosis in hypoxia significantly increased to 26% and dead cells increased to 13% as compared with normoxia of 7% and 2 % respectively (Figure 2a,b). The live cells in normoxia were 91% and reduced to 61% in hypoxia, reaching a total of 67% cells injury. This is consistent with published works [25,29,30]. Transfection of MC did not induce any additional changes (Figure 2b). Compared with MC, Let-7d-3p significantly increased live cells by 12% (61% to 69%), decreased early apoptosis cells by 67% (3% to 2%), late apoptosis cells by 78% (23% to 18%), and total apoptosis by 77% (26% to 20%). We also investigated the role of autophagy in the protective effect in both cells. Autophagy flux was assessed by the ratio of LC3 II/I with Bafilomycin A1 (Baf) treatment. Our data showed that Baf significantly increased LC3 II or LC3 II/I, an indication of autophagosome accumulation. This data is consistent with our published data [25]. However, Let-7d-3p could not reduce LC3 II/I with or without Baf under hypoxic conditions (Figure 3).

### 2.3. Let-7d-3p Target Gene Prediction and Validation

**Figure 4:**
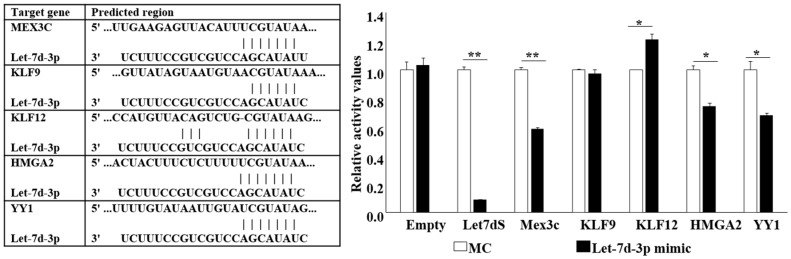
Validation of the gene targets of Let-7d-3p using luciferase assay. The predicted binding site of the target genes are shown in the Table. Let-7d-3p mimic reduced the luciferase activity of the Mex3c. HMGA2, and YY1 3’UTR constructs. Let-7d sensor (Let-7dS) served as a positive control. Data are presented as Mean ± SD with Student’s t-test, * *p* < 0.05, ** *p* < 0.01.

Let-7d-3p predicted gene targets were obtained from miRDB, TargetScan and miRWalk database. Five predicted target genes, HMGA2, KLF9, KLF12, MEX3C, and YY1 were selected as overlapping prediction between human and rat. The 3’-UTR of these genes were cloned for Luciferase (Luc) assay. Hela cells containing the target gene 3’UTR-Luc were treated with Let-7d-3p mimic. Let-7d sensor, a Let-7d complimentary sequence (Let-7dS), was used as positive control. A reduction of luciferase activity was observed in Mex3c, HMGA2, and YY1 indicating the interaction between Let-7d-3p and their 3’UTR (Figure 4).

### 2.4. Let-7d-3p Inhibits Apoptosis through Targeting HMGA2 and YY1

**Figure 5:**
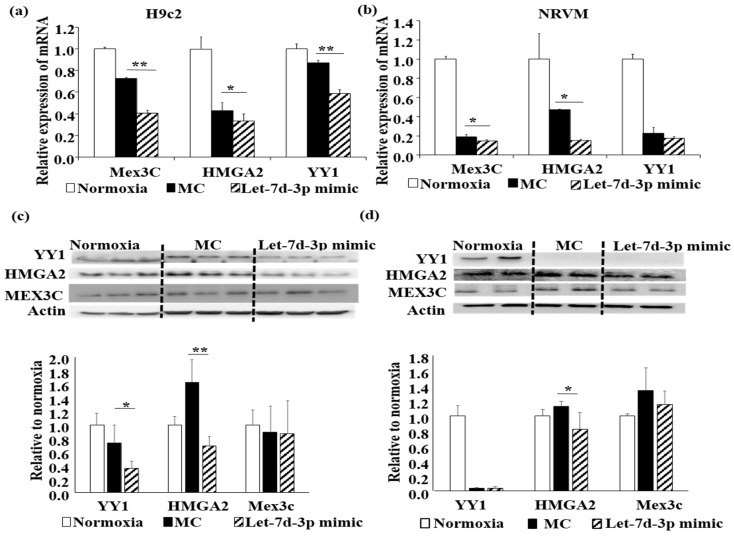
Verification of Let-7d-3p target genes. RT-qPCR measurements show the expressions of (**a**) Mex3c, HMGA2 and YYI were reduced in H9c2. (**b**) Mex3c and HMGA2 were reduced in NRVM. Western blot indicated that the protein level of (**c**) YY1 and HMGA2 were reduced in H9c2. (**d**) HMGA2 was reduced in NRVM. Experiments were repeated at least 3 times in triplicate. Data are presented as Mean ± SD with Student’s t-test, * *p* < 0.05, ** *p* < 0.01.

To further verify target gene regulation, gene expression was assessed by RT-qPCR and Western blot. Let-7d-3p reduced mRNA for Mex3c, HMGA2, and YYI in H9c2 (Figure 5a) and Mex3c and HMGA2 in NRVM (Figure 5b). Downregulation of YY1 and HMGA2 proteins was confirmed in H9c2 (Figure 5c). In NRVM, HMGA2 protein was significantly downregulated by Let-7d-3p (Figure 5d). The expressions YY1 both mRNA and protein was greatly reduced by hypoxia in NRVM without further down-regulation by Let-7d-3p. Taken together all the analyses indicated that Let-7d-3p protects cells undergoing hypoxic challenge, at least in part, through direct targeting of HMGA2. YY1 may be another direct target of Let-7d-3p.

## 3. Discussion

Our data suggests that Let-7d-3p exerts protective effects in both H9c2 and NRVM cells subjected to hypoxia. The protective effects are due to the inhibition of apoptosis. Targeting of HMGA2 through 3’-UTR direct binding may be the mechanism of this inhibition. Our current finding provides insight into the amelioration of hypoxia injury by Let-7d-3p and identifies Let-7d-3p as a potential therapeutic target in cardiac ischaemia and heart failure.

The level of Let-7d-3p is highly upregulated in clinical heart failure cohorts as compared with non-heart failure controls [5]. Upregulation of Let-7d-3p with mimic transfection protected H9c2 and NRVM against hypoxia injury. In contrast, down-regulation of Let-7d-3p had no effect. Coincidently, reversing the decreases of miR-374b-5p and miR-30b-5p with mimics yielded no beneficial effects. One of the challenges in the development of miRNA mediated therapeutics for the treatment of cardiovascular diseases is to identify such miRNA targets. To counteract significantly dysregulated miRNAs is a well-used stratagem. For example, pressure overload down-regulates miR-101a which activates TGF-β signaling in the heart. Reversal of the decrease in endogenous miR-101a with a mimic is beneficially anti-fibrotic [31]. However, the dysregulation of miRNA may not always be detrimental to a disease state. It could be a compensatory change. For example miR-221 is elevated in heart failure patients [5] and the upregulation of miR-221 exerts a protective effect in hypoxia-reoxygenation injury via targeting on Ddit4 and Tp53inp1 [25]. By performing functional screening on a set of dysregulated circulating miRNAs in heart failure patients, we subsequently found overexpression of Let-7d-3p protected H9c2 and NRVM against ischemic injury.

Ischemia-induced apoptosis and autophagy play an important role in ischemic injury. With 7-AAD and Anexin V double staining, Let-7d-3p inhibited early and late apoptosis suggesting Let-7d-3p can promote cell survival. Thus far, there has been no such report to show the protective effect of Let-7d-3p in this context and it has not even been characterized in any cardiovascular diseases. In ovarian cancer, the inhibition of Let-7d-3p using antagomiRs showed a significant decreased in cell proliferation and activated apoptosis in ovarian cancer cells [32]. These results seem in line with our finding that Let-7d-3p mimic enhanced cell viability and reduced apoptosis. Autophagy was assessed with the ratio of LC3 II/I and autophagy flux was assessed with Baf treatment, which blocks the autophagosome–lysosome fusion. The increase of LC3 II/I is an indication of the increased autophagosome formation. Let-7d-3p did not induce any changes of LC3 II nor the ratio of LC3 II/I with or without Baf. Therefore, Let-7d-3p did not affect autophagy in this context.

We further explored the underlying mechanisms of Let-7d-3p. Taking the conventional approaches of miRNA target prediction and validation, five relevant target genes were examined; Three of them, HMGA2, YY1, and Mex3c, were confirmed to have direct interaction with Let-7d-3p. Among them, HMGA2 and YY1 were downregulated at mRNA and/or protein levels, but Mex3c was not significantly downregulated at protein level in both cell types. Mex3c, a RNA-binding protein, plays a role in postnatal growth [33] and regulates energy balance [34]. It has no known effect in cardiac protection. YY1 protein expression was unchanged by hypoxia, but significantly reduced by Let-7d-3p in H9c2. Unlike H9c2, the YY1 protein level was completely suppressed in NRVM under hypoxia and thus Let-7d-3p mimic could not induce any further downregulation. YY1 is a transcriptional factor and plays a pivotal role in early heart development [35]. Its expression was elevated in the human failing heart, and caused pathologic hypertrophy [36]. The role of YY1 in ischemia is controversial. It is an important p53 negative regulator, therefore, inactivation of YY1 causes the cells to sensitize to DNA damage-induced apoptosis [37,38]. On the other hand, the inhibition of YY1 reduced the accumulation of hypoxia-inducible factor 1α (HIF1α) in a p53 independent manner [39]. Our data showed that its protein expression was unchanged in H9c2 but greatly reduced in NRVM by ischemia. The reason for this cell type specific reaction is unclear. Taken together, we suspect that YY1 regulation may not play a role in this setting. The protein expression of HMGA2 was significantly upregulated in hypoxia and Let-7d-3p suppressed this change in both H9c2 and NRVM. This data render it a promising target for Let-7d-3p induced protective effects.

HMGA2 has been widely known as an oncoprotein in cancer biology [40]. There have been strong evidences shown that HMGA2 is an important trigger of apoptosis in cancer research. The damage of double-strand DNA is dependent on the non-homologous end joining (NHEJ) process to repair [41]. HMGA2 blocks this repair through the inhibition of DNA-dependent protein kinase (DNA-PK) [42]. Therefore, the increase of HMGA2 will lead to the accumulation of cells with DNA-damage. It has been further supported by a study showing that HMGA2 overexpression may initialize caspase-2 activation and further induce downstream caspase-3 release, a marker of mitochondrial mediated apoptosis [28]. They also demonstrated that HMGA2-induced apoptosis is correlated with the up-regulation of cleaved caspase 3, suggesting that the HMGA2-induced apoptosis is dependent on the pathway of DNA damage. It is known that ischemia-induced DNA damage is a trigger of ischemic apoptosis [17]. Therefore, it is reasonable to speculate that the upregulation of HMGA2 blocks repair of damaged DNA in ischemia, which in turn causes apoptosis. Downregulation of HMGA2 by Let-7d-3p is beneficial. Further evidence confirms that the pro-apoptotic effects of HMGA2 are caused by the upregulation of p53, Bax, cleaved caspase 9 and down regulation of anti-apoptotic gene Bcl-2 and Apaf1 [28]. One early work showed that Let-7a suppresses HMGA2 expression in cancer cells [43]. As the Let-7 family share same seed region sequence, this supports our finding. Taken together, through direct targeting on HMGA2, Let-7d-3p inhibited apoptosis and protected the cells against ischemia injury.

Apoptosis was only assessed in H9c2, as NRVM are susceptible to trypsin digestion not suitable for flow-based Annexin V/7-AAD assay. Except for this, all experiments were carried out in parallel in H9c2 and NRVM. The results are highly consistent. In summary, screening a pre-selected set of miRNAs from our previous clinical study, we found that increasing the expression of Let-7d-3p reduced cell injury and apoptosis. Taking the approaches of luciferase reporter assay, RT-qPCR and Western blot, we demonstrated that HMGA2 is a direct target of Let-7d-3p. Supported by published results, we speculated that the anti-apoptotic effect of Let-7d-3p in hypoxia is due to the downregulations of HMGA2.

## 4. Materials and Methods

### 4.1. H9c2, NRVM Isolation and Culture

H9c2 cells, a rat cardiomyoblast cell line were grown in Dulbecco’s modified eagle medium (DMEM) containing 10% fetal bovine serum (FBS) and 1% of penicillin-streptomycin at 37 °C under an atmosphere of 95% air and 5% CO_2_. NRVMs were isolated from 1–3 day old neonatal rats by enzymatic digestion and mechanical dissociation as described previously [25]. Purified cardiomyocytes were seeded in collagen coated dish with a 1:4 of M199 and DMEM containing 15% FBS and 1% antimycotic antibiotics. After overnight attachment, the culture medium was replaced by a 1:4 of M199 and DMEM containing 10% FBS, 50µM cytosine arabinoside-C, and 1% antimycotic antibiotics and maintained throughout cultures.

### 4.2. miRNA Transfection

MiRNA mimics (miRVana, Ambion, Singapore) were transfected in H9c2 and NRVM according to the manufacturer’s instructions. Briefly, a final concentration of 25 nM of miRNA mimics and lipofectamine RNAiMAX (Invitrogen, Singapore) was incubated at a ratio of 20 pmol:1 µL. Mimic control (MC), Let-7d-3p inhibitor and inhibitor control (IC) were transfected under the same conditions [29].

### 4.3. Hypoxia Protocol

Following 24 h of transfection, H9c2 was subjected to 15 h of hypoxia (0.2% oxygen) with serum free DMEM containing 1% glutamax and 1% of penicillin-streptomycin. NRVM was subjected to 6 h of hypoxia (0.2% Oxygen) in modified Esumi buffer (137 nM NaCl, 12 mM KCl, 0.5 mM MgCl2, 0.9 mM CaCl2, 4 mM Hydroxyethyl piperazineethanrsulfonic acid (HEPES) sodium salt, 20 mM sodium lactate, 10 mM 2-deoxyglucse, pH 6.2). For the normoxia group, cells were cultured in culture medium under normal conditions [25,29].

### 4.4. Assessment of Cell Injury and Viability

After hypoxia treatment, lactate dehydrogenase (LDH) release was measured using cytotoxicity detection kit (TOX7, Sigma-Aldrich Corp., Singapore) according to the manufacture instruction. The absorbance of the samples was measured at 490 nm (EnSpire multimode plate reader, Perkin Elmer, Waltham, MA, USA). The viability of cells, CCK8 (Sigma-Aldrich Corp., Singapore) was measured according to manufacturer’s instructions. The absorbance of the samples was measured at 460 nm.

### 4.5. Assesment of Apoptosis and Autophagy

H9c2 were trypsinized for the assessment of apoptosis using Annexin V and 7-AAD double staining (MUSE cell analyzer, Merck Millipore Corp, Singapore) according to the manufacturer’s instruction. For autophagy analysis, cells were treated with either DMSO or 10nM Baf A1 for 2 h after hypoxia. Cells were lysed for Western blot analysis against LC-3 I and II (SC-376404, Santa Cruz Biotechnology, Axil Scientific, Singapore). β-actin (SC-47778, Santa Cruz) was used as a loading control.

### 4.6. Luciferase Assay

The 3′UTR of target genes were directionally cloned into the psiCHECK-2 vectors (Promega, Corp., Singapore). The plasmid and Let-7d-3p mimics were co-transfected into Hela cells. The luciferase activity in cell lysate was determined using the Dual-Luciferase Reporter Assay System (DLR assay system, Promega Corp., Singapore) according to the manufacturer’s protocol. Let-7d-3p sensor (Let-7dS), a complementary binding sequence of Let-7d-3p, was used as a positive control.

### 4.7. Real-time Quantitative PCR (RT-qPCR) Analysis

Total RNAs from H9c2 and NRVM were extracted by Trizol reagent (Invitrogen, Singapore) according to the manufacturer’s protocol. Primers (Integrated DNA Technologies, Singapore) used to detect the mRNA expressions were HMGA2 (Rn.PT.58.44563776), YY1 (Rn.PT.58.7943099), MEX3C (Rn.PT.58.9714240), and endogenous control RPLP0 (Rn.PT.58.32850825). Gene expressions were measured by universal reverse transcription and RT-qPCR (Quant Studio™ 7 Flex Real-Time PCR system, Applied Biosystems, Singapore) and quantified using the 2^−∆∆Ct^ method.

### 4.8. Western Blot

An equal amount of denatured protein sample was resolved by 10% SDS-polyacrylamide gel, transferred to PVDF membranes and probed with antibodies against YY1, HMGA2, Mex3C, and β-actin (SC-7341, SC-30223, SC-398440, SC-47778, Santa Cruz). After incubating with peroxidase-conjugated secondary antibody (BioRad, Singapore) and ECL blotting substrate (BioRad, Singapore), the protein of interests were visualized by the G:Box gel doc system (Chemi XT4, Syngene, Frederick, MD, USA).

### 4.9. Statistics

All data were expressed as Mean ± SD. Student’s *t*-test was performed to compare the difference between the control and treated group. *p* < 0.05 was used as the criterion for statistical significance.

## Figures and Tables

**Table 1:** Selected miRNA from clinical discovery data.

Name	Human Sequence	Rat Sequence
Let-7d-3p	C**UAUACG**ACCUGCUGCCUUUCU	C**UAUACG**ACCUGCUGCCUUUCU
miR-503-5p	U**AGCAGC**GGGAACAGUUCUGCAG	U**AGCAGC**GGGAACAGUACUGCAG
miR-134-5p	U**GUGACU**GGUUGACCAGAGGGG	U**GUGACU**GGUUGACCAGAGGGG
miR-374b-5p	A**UAUAAU**ACAACCUGCUAAGUG	A**UAUAAU**ACAACCUGCUAAGUG
miR-30b-5p	U**GUAAACA**UCCUACACUCAGCU	U**GUAAAC**AUCCUACACUCAGCU
